# Outbreak of African Swine Fever, Vietnam, 2019

**DOI:** 10.3201/eid2507.190303

**Published:** 2019-07

**Authors:** Van Phan Le, Dae Gwin Jeong, Sun-Woo Yoon, Hye-Min Kwon, Thi Bich Ngoc Trinh, Thi Lan Nguyen, Thi To Nga Bui, Jinsik Oh, Joon Bae Kim, Kwang Myun Cheong, Nguyen Van Tuyen, Eunhye Bae, Thi Thu Hang Vu, Minjoo Yeom, Woonsung Na, Daesub Song

**Affiliations:** Vietnam National University of Agriculture, Hanoi, Vietnam (V.P. Le, T.B.N. Trinh, T.L. Nguyen, T.T.N. Bui);; Korea Research Institute of Bioscience and Biotechnology, Daejeon,; South Korea (D.G. Jeong, S.-W. Yoon, H.-M. Kwon);; Median Diagnostics, Chuncheon-si, South Korea (J. Oh, J.B. Kim, K.M. Cheong);; Gold Coin, Hai Duong, Vietnam (N.V. Tuyen);; Korea University, Sejong, South Korea (E. Bae, T.T.H. Vu, M. Yeom, D. Song);; Chonnam National University, Gwangju, South Korea (W. Na)

**Keywords:** African swine fever, Vietnam, viruses, swine, outbreak

## Abstract

African swine fever is one of the most dangerous diseases of swine. We confirmed the 2019 outbreak in Vietnam by real-time reverse transcription PCR. The causative strain belonged to p72 genotype II and was 100% identical with viruses isolated in China (2018) and Georgia (2007). International prevention and control collaboration is needed.

Since its first identification in East Africa in the early 1900s, African swine fever (ASF) spread to Kenya in the 1920s; transcontinental outbreaks in Europe and South America in the 1960s and in Georgia (Caucasus) in 2007 led to subsequent transmission to neighboring countries east of Georgia. Along with the outbreaks in the eastern territory of the Russian Federation, acute ASF outbreaks were reported in China in 2018 (*1*).

During January 15–31, 2019, a disease outbreak at a family-owned backyard pig farm in Hung Yen Province, Vietnam, was reported. The farm, ≈50 km from Hanoi and 250 km from the China border, housed 20 sows. In the early stage of the outbreak, 1 piglet and 1 sow exhibited marked redness all over the body, conjunctivitis, and hemorrhagic diarrhea. Breeding gilts demonstrated anorexia, cyanosis, and fever (>40.5°C). 

On February 1, 2019, after confirming that the mortality rate at this farm had surpassed 50%, we collected organ samples (e.g., spleen, liver, kidney, tonsil, and lymph nodes) from dying pigs and submitted them to the diagnostic laboratory at the Vietnam National University of Agriculture for ASF diagnosis. All specimens underwent homogenization, followed by extraction of viral DNA (*2*). ASF virus DNA was identified by routine PCR, as recommended by the Office International des Epizooties (Paris, France), and by commercialized real-time PCR (Median Diagnostics Inc., http://www.mediandiagnostics.com). We named the detected ASF virus VNUA/HY-ASF1 and deposited the following complete genome sequences into GenBank: p10 (accession no. MK795932), p11.5 (MK795933), p12 (MK795934), p14.5 (MK795935), p17 (MK795936), p22 (MK795937), pE248R (MK795938), p30 (MK757460), p54 (MK554697), p72 (MK554698), and Cd2v (MK757459). We aligned the nucleotide sequences by using BioEdit version 7.2 (Ibis Biosciences, http://www.mbio.ncsu.edu/bioedit/bioedit.html) with ClustalW (http://clustal.org) and calculated sequence identity. Using MEGA7 (https://www.megasoftware.net)and the neighbor-joining method, we based phylogenetic analysis on the genetic information and calculated bootstrap values with 1,000 replicates. The genotype was determined by p72 gene characterization as reported previously (*3*,*4*). Phylogenetic trees revealed that the VNUA/HY-ASF1 strain belonged to p72 genotype II (Figure) and was 100% identical to China strains SY18/China/2018 (GenBank accession no. MH713612) and AnhuiXCGQ/China/2018 (MK128995) and other genotype II strains of Europe: Georgia/2007/1 (GenBank accession no. FR682468.1), Russia/2012 (KJ195685), Estonia/2014 (LS478113), and Poland/2015 (MH681419).

**Figure:**
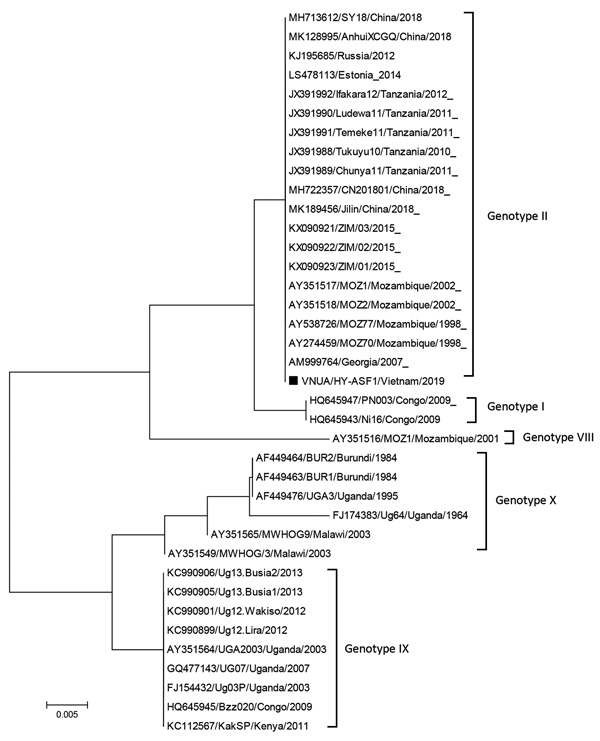
Phylogenetic analysis of major capsid protein gene (p72) of African swine fever virus isolated during outbreak in Vietnam in 2019 (VNUA HY-ASF1; black square) and reference isolates. The phylogenetic tree was constructed by using the neighbor-joining method in MEGA7 (http://www.megasoftware.net). Bootstrap values were calculated with 1,000 replicates. GenBank accession numbers, strain name, country, and year of collection are indicated. Scale bars indicate nucleotide substitutions per site.

The clinical signs and necropsy findings of the pigs involved in the 2019 outbreak in Vietnam were similar to those caused by the virus strains in China and Georgia (e.g., high mortality rates over a short period and multifocal hemorrhagic lesions in many organs). However, the clinical forms and pathophysiology of ASF varied according to virus virulence, exposure dose, and transmission route.

Considering the epidemiologic features of the site where ASF has recently occurred, the virus is highly likely to have reached Vietnam via infected wild boar, by movement of pigs and pork products, or by infected fomites (*5*). The most probable source and major cause of transmission across the countries is thought to be ASF virus–contaminated pork products (*2*). The outbreak in Vietnam was confirmed in the northern part of the country, near China, where many instances of illegal movement of animals and meat products across the China–Vietnam border have been reported (http://www.fao.org/3/i8805en/I8805EN.pdf). Therefore, it is likely that the virus originated in China.

Although the p30, p54, and p72 sequences were 100% identical to those from China and Georgia, whole genomes must be monitored for possible changes and further spread of the ASF virus. Since the 2018 outbreak in China, the subsequent ASF outbreak in Vietnam (February 1, 2019) increases the possibility of virus spread to nearby swine-raising Southeast Asia countries, including Laos, Thailand, Cambodia, and Myanmar. Although ASF has occurred in many countries, including Russia and Europe, its outbreak in Asia is far more critical because 60% of the world’s pig population is concentrated in that area and the socioeconomic effects of swine disease would be greater than that in other regions. Therefore, to avoid great economic losses worldwide, we highly recommend that preventive and control measures be developed and implemented through international collaboration.
